# Gut Microbiota’s role in lipoma development: evidence from mendelian randomization

**DOI:** 10.3389/fgene.2024.1430671

**Published:** 2024-11-15

**Authors:** Yuxin Li, Jiahao Chen, Hang Yao, Xiaogang Xu, Xianglong Zheng, Yu Wang, Wanchun Wang

**Affiliations:** ^1^ Graduate School of Jiangxi University of Traditional Chinese Medicine, Nanchang, China; ^2^ School of Basic Medical Sciences, Zhejiang Chinese Medical University, Hangzhou, China; ^3^ School of Traditional Chinese Medicine, Binzhou Medical University, Yantai, China; ^4^ Department of Traditional Chinese Medicine Surgery, Affiliated Hospital of Jiangxi University of Traditional Chinese Medicine, Nanchang, China

**Keywords:** gut microbiota, causal relationship, lipoma, mendelian randomization analysis, tumour microenvironments

## Abstract

**Background:**

Lipoma, a benign tumor derived from mesenchymal tissue, significantly affects patients’ physical and psychological wellbeing. Increasing evidence points to a strong link between the gut microbiome (GM) and lipoma incidence. This study utilizes Mendelian Randomization (MR) to assess the potential causal relationships between the GM and lipoma development.

**Methods:**

We conducted a two-sample MR analysis using genome-wide association study (GWAS) data from MiBioGen and FinnGen to explore the causal relationship between GM and lipoma. The GM dataset included 18,340 participants with 14,587 single nucleotide polymorphisms (SNPs), while the lipoma dataset comprised 412,181 participants with 21,306,349 SNPs. We employed 5 MR methods: Inverse Variance Weighted (IVW), Weighted Median, Simple Mode, MR-Egger, and Weighted Mode. Additional assessments included Cochran’s Q test for result heterogeneity, PRESSO analysis for horizontal pleiotropy, and sensitivity analyses through scatter plots, leave-one-out analyses, funnel plots, and forest plots.

**Results:**

The IVW method identified 18 gene predictors trans-genus associated with lipoma risk. Protective effects against benign lipoma (BL) were observed in the Eubacterium rectale group, Desulfovibrio, Ruminococcus1, *Clostridium* sensu stricto1, and Lachnospiraceae UCG001; conversely, Lachnospiraceae UCG008 was linked to increased BL risk. Desulfovibrio provided protection against TS-BL; however, the Family XIII AD3011 group, Eubacterium coprostanoligenes group, Lachnospiraceae NK4A136 group, and Parasutterella were associated with an increased TS-BL risk. The *Clostridium* innocuum group, Eubacterium rectale group, Anaerotruncus, Ruminiclostridium6, and Lachnospiraceae UCG001 offered protection against LS-BL, while Lachnospiraceae UCG008 was linked to an increased LS-BL risk. The Eubacterium brachy group, Odoribacter, Butyricimonas, Subdoligranulum, and *Clostridium* sensu stricto1 were protective against HFNS-BL; Ruminococcaceae UCG005 was associated with an increased HFNS-BL risk.

**Conclusion:**

Compared to malignant tumors, research on lipomas has been relatively limited. This study, through MR analysis, provided new evidence of a causal relationship between specific GM and the development of lipomas. Certain gut bacterial species may act as protective or harmful factors in lipoma formation, offering new avenues for future treatment strategies. However, additional research is required to unravel the complexity of how GM influences the pathogenesis of lipomas.

## 1 Introduction

Lipoma is a prevalent subcutaneous benign tumor arising from adipose tissue, typically surrounded by a thin fibrous tissue. Variants such as Benign Lipoma (BL), and BLs affecting skin and subcutaneous tissues of the trunk (TS-BL), limbs (LS-BL), and head, face, and neck (HFNS-BL) are common (Kosztyuova and Shim, 2017). Lipomas may occur not only superficially but also within deep soft tissues, especially in the back, upper limbs, and thighs, where they predominantly exhibit an infiltrative growth pattern (Inamura et al., 2021; Ameloot et al., 2022). Rare variants may also appear in muscles, within joints, or bronchi (Seo et al., 2018; Wang et al., 2019; Nader et al., 2012), manifesting as either solitary or multiple lesions at any age. Studies suggest that genetic predisposition and external factors such as ionizing radiation, chlorophenol, and viruses may contribute to lipoma development (Palitot DE Melo et al., 2022). Currently, lipomas and their subtypes constitute over half of all soft tissue tumors, with a prevalence rate of 2.1% (Marteau et al., 2020), and approximately 5% of cases present as multiple lipomatosis (Charifa et al., 2024). Furthermore, traditional treatments, including surgical removal and vacuum aspiration, face challenges due to high recurrence rates and difficulties in complete removal, underscoring the importance of understanding their pathological characteristics and exploring potential treatment options (Pereira and Schonauer, 2001). Lipoma remains one of the most commonly encountered soft tissue tumors in clinical practice, yet its precise causes and underlying mechanisms, particularly its potential associations with the GM, are not well-understood. Therefore, a deeper investigation into the pathological mechanisms of lipomas, especially their relationship with the microbiome, is crucial for elucidating their etiology and developing new therapeutic approaches.

Compelling evidence suggests that the GM plays a role in the regulation of metabolic disorders, psychiatric conditions, and cancers, including gout (Tong et al., 2022), Alzheimer’s disease (Varesi et al., 2022), and lymphoma (Ml and Rh, 2014). Recent findings indicate that microbiome-induced tumors in the gastrointestinal tract and uterus are well-documented. As part of the tumor microenvironment (TME), the GM may influence the formation and progression of lipomas (Kovács et al., 2020). Dysbiosis of the GM can affect systemic immune functions, compromise mucosal barriers, and modulate immune responses that may promote or inhibit tumor development (Cs and Mj, 2011; Kawai and Akira, 2009). Additionally, moderate gut dysbiosis observed in lipoma patients can lead to reduced microbial diversity (Tkachuk et al., 2023).

Given the costs, time constraints, and ethical considerations associated with clinical trials, Mendelian Randomization (MR) has emerged as a robust method (Song et al., 2024). Commonly used to examine causal relationships between exposures and outcomes, MR employs single nucleotide polymorphisms (SNPs) derived from genome-wide association study (GWAS) as instrumental variables to establish causality. This method has been utilized to explore potential causal links between the GM and various diseases (Sekula et al., 2016). Based on existing research, this study hypothesized that an imbalance in the GM is causally related to the development of lipomas. By applying MR analysis, we aimed to uncover the potential role of the GM in lipoma formation, providing new insights into the pathological mechanisms underlying lipomas.

## 2 Materials and methods

### 2.1 Study overview

**FIGURE 1:**
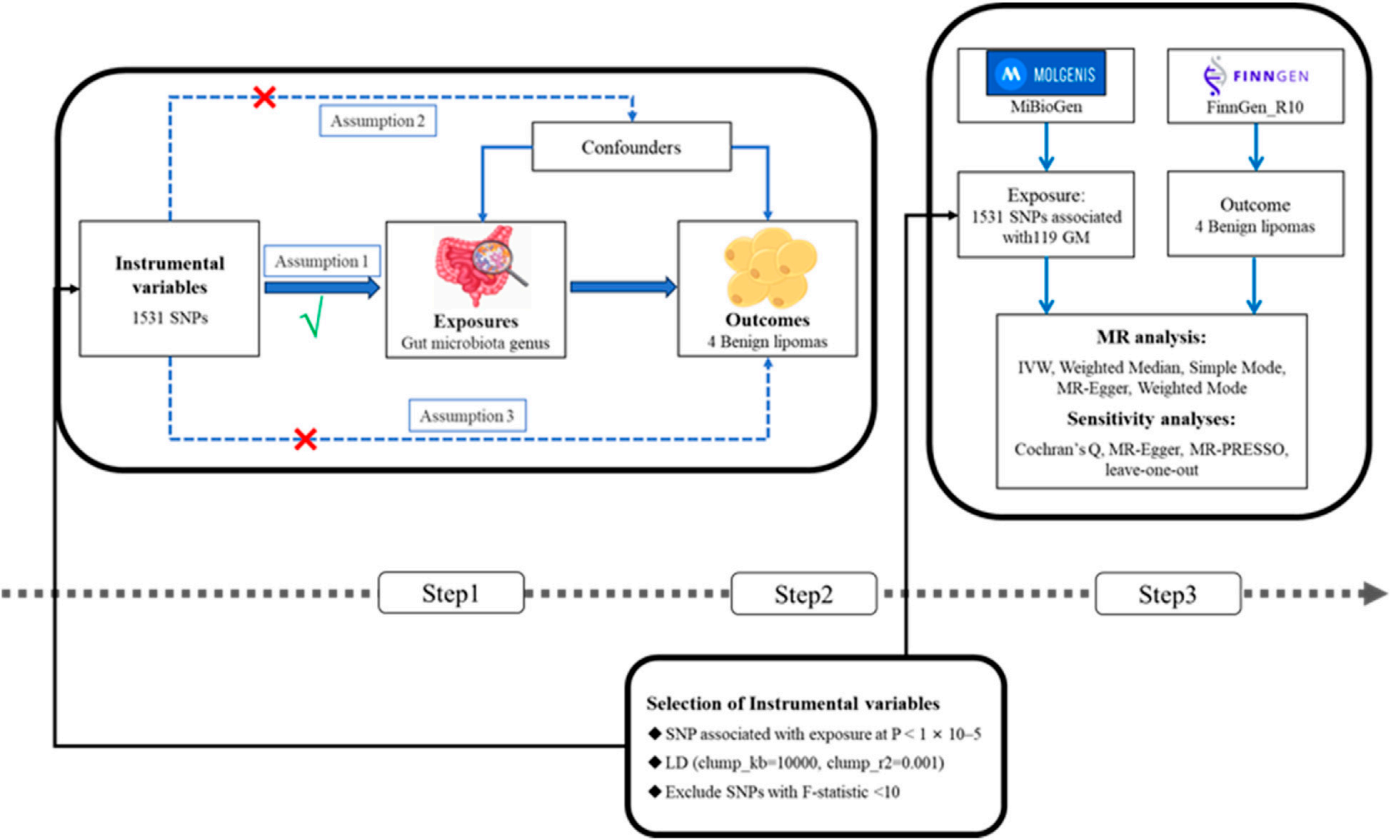
(Step1) Three assumptions of MR. I, Correlation assumption; II, independence assumption; III, exclusionary restriction assumption; (Step2) Selection of instrumental variables; (Step3) Flowchart of this MR study. MR, Mendelian randomization; SNP, single nucleotide polymorphism.

This research conducted a two-sample MR analysis to explore the causal relationships between the GM and four types of lipomas. Comprehensive GWAS data were obtained for both the GM and lipomas. Each genus level of the GM was considered an independent exposure factor, with BLs and three other location-specific lipomas as outcome factors. To ensure the reliability of our findings, this MR study adhered to three critical assumptions: 1) SNPs, used as instrumental variables (IVs), are closely associated with the exposure (GM); 2) SNPs are independent of confounding factors; 3) SNPs affect lipoma risk solely through the GM and not through other pathways (VanderWeele et al., 2014; Liu Y. et al., 2022). The workflow of this study is depicted in Figure 1.

### 2.2 Data sources

The GWAS data for the GM were initially sourced from the MiBioGen consortium, involving a large-scale, ethnically diverse analysis of 18,340 individuals (Kurilshikov et al., 2021). We excluded 12 genera with unknown classifications, ultimately analyzing 119 genera (Xia et al., 2023). Additionally, GWAS data for BL, TS-BL, LS-BL, and HFNS-BL were obtained from the FinnGen R10 database (FinnGen provides genetic insights from), with all data derived from European populations. All summary data utilized in this study are publicly available and have received ethical approval from their respective institutions (Table 1).

**TABLE 1:** Details of the GWASs included in the Mendelian Randomization.

Trait	Data type	N_cases	N_controls	Consortium
Gut Microbiota	Exposure	18,340		MiBioGen
BL	Outcome	10,276	401,905	FinnGen_R10
TS-BL	Outcome	4,094	408,087	FinnGen_R10
LS-BL	Outcome	2,344	409,837	FinnGen_R10
HFNS-BL	Outcome	1,909	410,272	FinnGen_R10

### 2.3 Selection of instrumental variables

To validate the accuracy of the causal relationship between the GM and lipomas, this study implemented rigorous quality control procedures. Initially, SNPs associated with specific genera were selected as IVs using a *p*-value threshold (*p* < 1 × 10^−5^). Linkage disequilibrium (LD) analysis was then conducted on a European genomic dataset (clump_kb = 10,000, clump_*r* ^2^ = 0.001) (Chen et al., 2023), excluding palindromic SNPs to mitigate allelic biases. The strength of the IVs was assessed by calculating the F-statistic, where an F > 10 indicated robust instruments and IVs with F ≤ 10 were excluded. The F-statistic was calculated as follows: F = β^2^_exposure/SE^2^_exposure (Wang et al., 2024).

### 2.4 Statistical methods and sensitivity analysis

Five MR analysis methods were utilized to thoroughly investigate the potential causal relationship between the GM and lipomas. These methods included inverse variance weighted (IVW), weighted median, simple mode, MR-Egger, and weighted mode, with IVW serving as the primary method (Lee et al., 2016). IVW, a commonly used approach, combines the effects of each genetic variant by weighting them inversely to their estimated uncertainty to maximize statistical efficiency. The weighted median method estimates causal effects while addressing pleiotropy, assigning weights to each IV, and estimating the causal effect as the weighted median. Simple mode estimates causal effects by selecting the most common effect direction among IVs, assuming a cluster around a true causal effect value. MR-Egger regression detects and adjusts for horizontal pleiotropy by allowing for pleiotropy among IVs and adjusting the causal effect estimate through the Egger regression approach, with the intercept testing for systematic pleiotropy. Lastly, the weighted mode method assigns weights to different IVs and integrates their effects, assuming the most significant IVs contribute the greatest weight and their mode represents the causal effect.

Cochran’s Q test assessed result heterogeneity, with a *p* < 0.05 indicating significant heterogeneity. Depending on heterogeneity, either a random-effects model or a fixed-effects model was applied. Horizontal pleiotropy was evaluated using MR-Egger and MR-PRESSO analyses, with an intercept *p* > 0.05 indicating no significant horizontal pleiotropy. A leave-one-out test was employed to identify individual SNPs with a significant impact on the MR analysis. Additionally, funnel plots and forest plots were utilized to enhance the robustness of the findings. All statistical analyses were conducted using Rstudio (version 4.3.2), utilizing the TwoSampleMR package.

## 3 Results

### 3.1 Selection of instrumental variables

**FIGURE 2:**
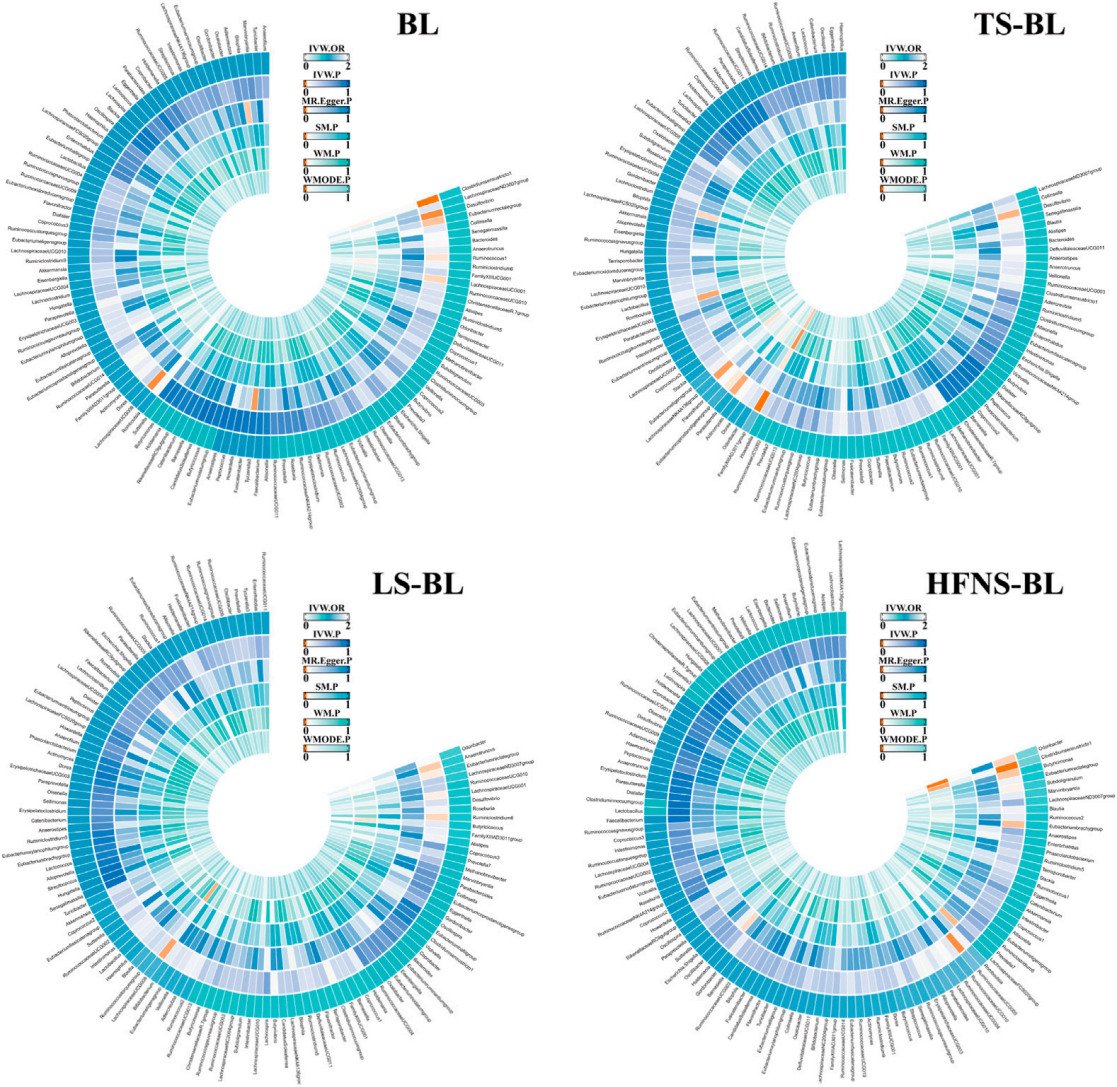
The circus plot showing the MR results of all gut microbiota. IVW, inverse-variance weighted; SM, Simple mode; WM, Weighted median; WMOED, Weighted mode; P, *p*-value; OR, odds ratio.

SNPs related to the GM were sourced from the MiBioGen database. After applying a *p*-value threshold (*p* < 1 × 10^−5^), a total of 14,587 SNPs associated with the GM were identified. These SNPs underwent further scrutiny through LD analysis. Utilizing the selection criteria for IVs, 1531 SNPs linked to 119 GM taxa were selected as IVs, all demonstrating F-statistics greater than 10 (Yao et al., 2024), suggesting a minimal likelihood of weak instrument bias. For more detailed information, (Supplementary Table S1). Figure 2 depicts the MR causal relationships between the 119 GM taxa and four types of lipomas, with separate results listed in Supplementary Tables S2–S5.

### 3.2 Causal relationship between GM and 4 types of lipoma

#### 3.2.1 Causal relationship between GM and BL

**FIGURE 3:**
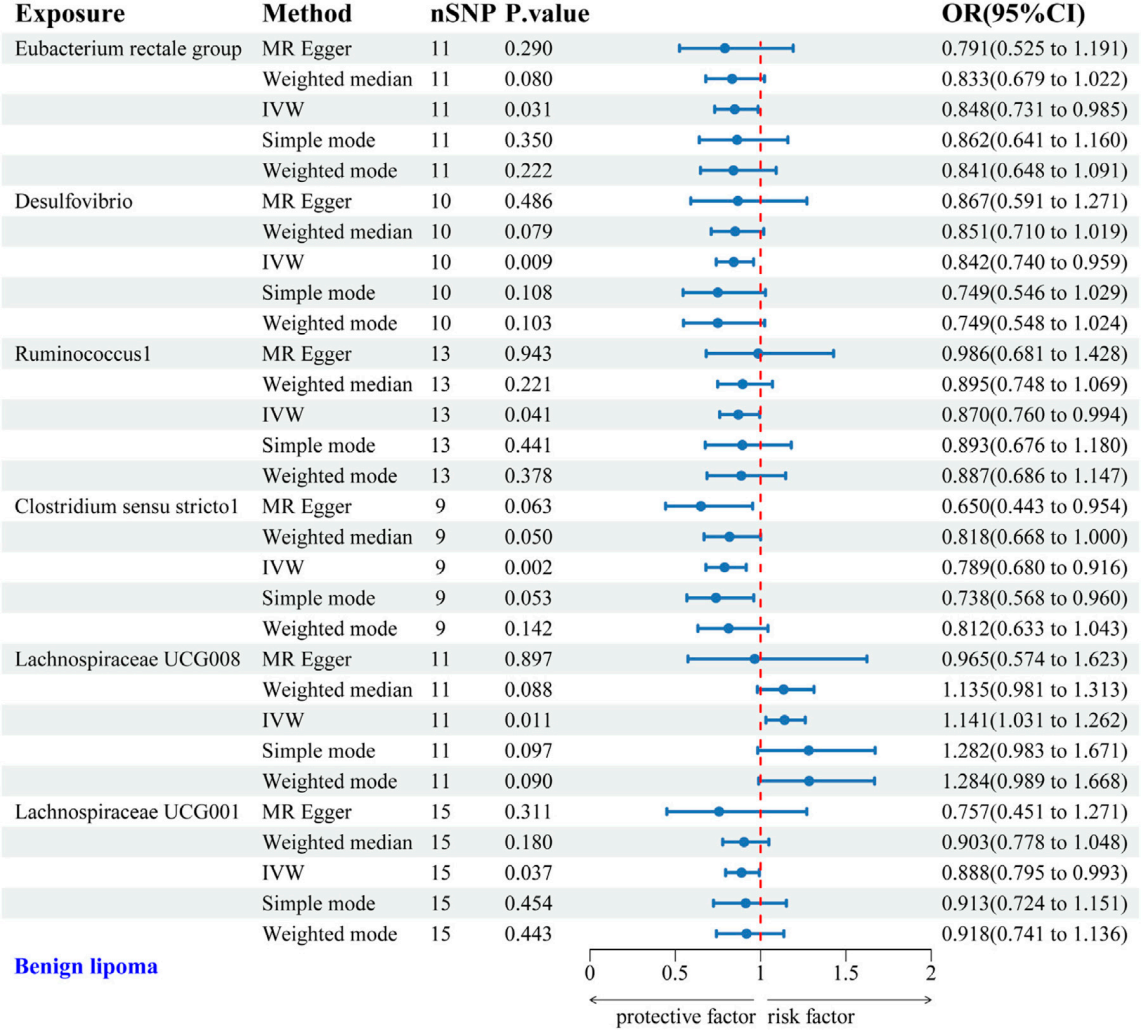
Forest plot of the associations between genetically predicted GM and BL risk using IVW methods. IVW, inverse-variance weighted. OR, odds ratio.

IVW analysis identified protective associations between six gut microbial genera and the risk of BL. Specifically, the Eubacterium rectale group (OR = 0.848, 95% CI: 0.731–0.985), Desulfovibrio (OR = 0.842, 95% CI: 0.740–0.959), Ruminococcus1 (OR = 0.870, 95% CI: 0.760–0.994), *Clostridium* sensu stricto1 (OR = 0.789, 95% CI: 0.680–0.916), and Lachnospiraceae UCG001 (OR = 0.888, 95% CI: 0.795–0.993) exhibited protective effects against BL. Conversely, Lachnospiraceae UCG008 (OR = 1.141, 95% CI: 1.031–1.262) was linked to an increased risk of BL (Figure 3). Scatter plots from the MR analysis displayed the estimated effects of GM SNPs on BL (Supplementary Figure 1A).

Results from Cochran’s Q, MR Egger, and MR-PRESSO analyses indicated no evidence of heterogeneity or horizontal pleiotropy among the six genera associated with BL (Table 2). Leave-one-out analysis, funnel plots, and forest plots confirmed the reliability of the MR findings (Supplementary Figures 1B, C).

**TABLE 2:** Sensitivity analysis of the MR analysis results of the GM and BL.

Exposure	Heterogeneity		Directional pleiotropy		MR-PRESSO
	Cochran’s Q	*p*-value	Egger intercept	*p*-value	*p*-value
Eubacterium rectale group	4.380	0.885	0.005	0.726	0.935
Desulfovibrio	6.977	0.539	−0.003	0.880	0.655
Ruminococcus1	6.090	0.867	−0.011	0.489	0.901
*Clostridium* sensu stricto1	5.918	0.549	0.020	0.318	0.591
Lachnospiraceae UCG008	8.958	0.441	0.018	0.537	0.527
Lachnospiraceae UCG001	15.275	0.290	0.015	0.545	0.361

#### 3.2.2 Causal relationship between GM and TS-BL

**FIGURE 4:**
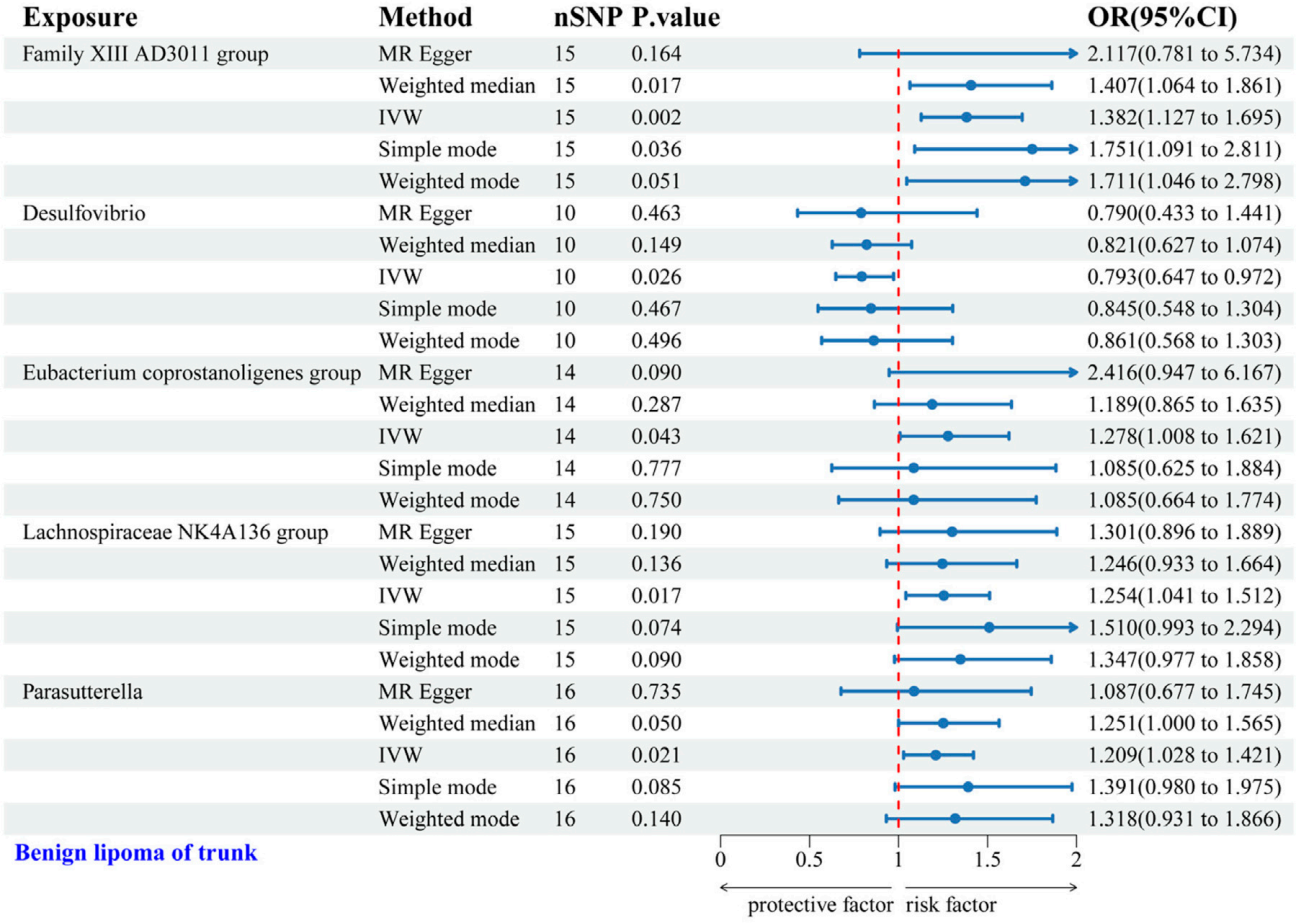
Forest plot of the associations between genetically predicted GM and TS-BL risk using IVW methods. IVW, inverse-variance weighted. OR, odds ratio.

IVW analysis revealed associations between five gut microbial genera and the risk of TS-BL. Desulfovibrio exhibited a protective effect against TS-BL (OR = 0.793, 95% CI: 0.647–0.972), whereas increased risks were associated with the Family XIII AD3011 group (OR = 1.382, 95% CI: 1.127–1.695), Eubacterium coprostanoligenes group (OR = 1.278, 95% CI: 1.008–1.621), Lachnospiraceae NK4A136 group (OR = 1.254, 95% CI: 1.041–1.512), and Parasutterella (OR = 1.209, 95% CI: 1.028–1.421) (Figure 4). Scatter plots from the MR analysis illustrate the estimated effects of GM SNPs on TS-BL (Supplementary Figure 2A).

Results from Cochran’s Q, MR Egger, and MR-PRESSO analyses revealed no significant heterogeneity or horizontal pleiotropy among the five genera related to TS-BL (Table 3). Leave-one-out analysis, funnel plots, and forest plots indicated the robustness of the MR study results (Supplementary Figures 2B, C).

**TABLE 3:** Sensitivity analysis of the MR analysis results of the GM and TS-BL.

Exposure	Heterogeneity		Directional pleiotropy		MR-PRESSO
	Cochran’s Q	*p*-value	Egger intercept	*p*-value	*p*-value
Family XIII AD3011 group	7.448	0.878	−0.035	0.407	0.897
Desulfovibrio	5.955	0.652	0.000	0.988	0.777
Eubacterium coprostanoligenes group	8.041	0.782	−0.041	0.194	0.716
Lachnospiraceae NK4A136 group	10.754	0.631	−0.003	0.828	0.731
Parasutterella	9.078	0.826	0.009	0.647	0.860

#### 3.2.3 Causal relationship between GM and LS-BL

**FIGURE 5:**
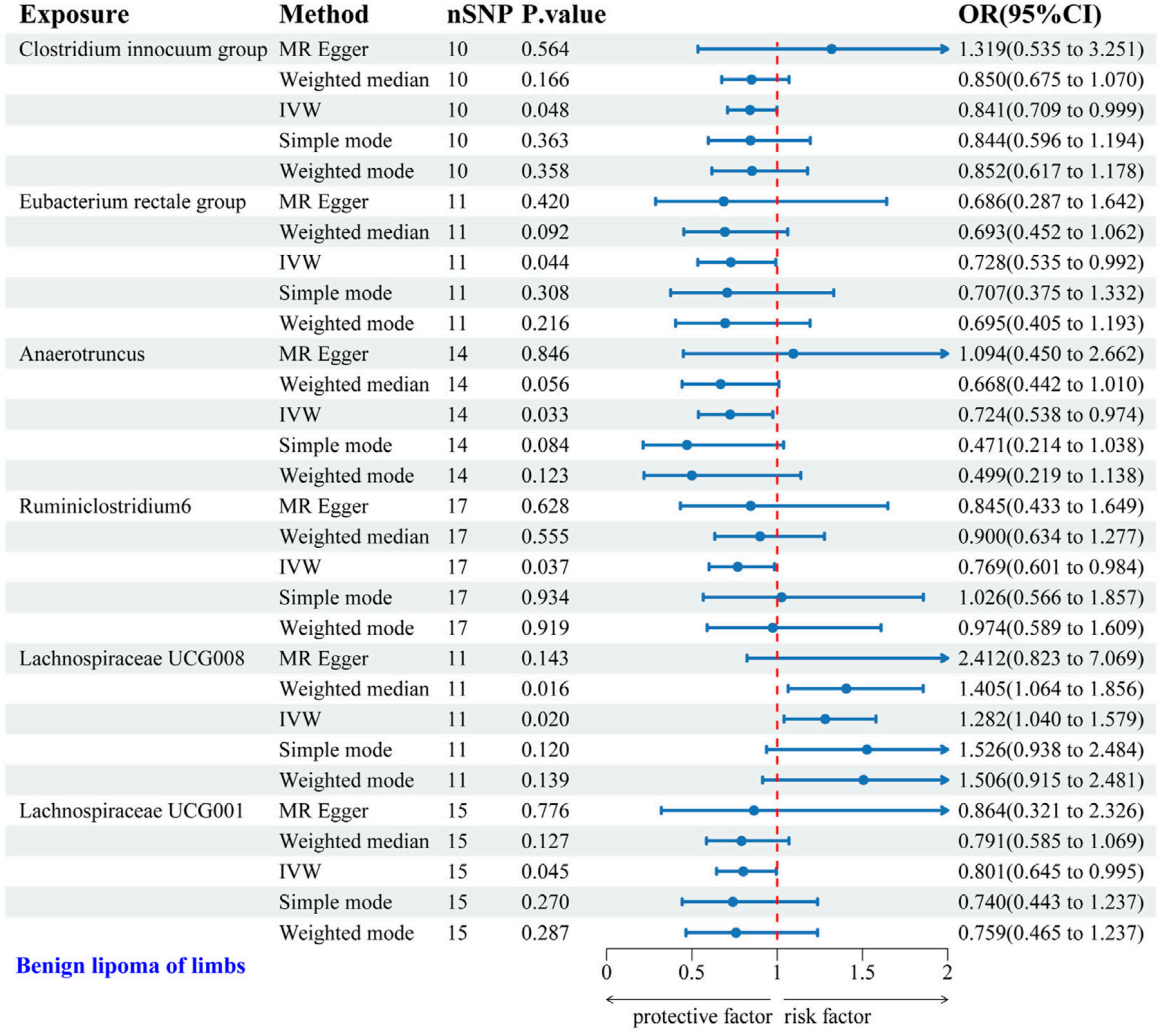
Forest plot of the associations between genetically predicted GM and LS-BL risk using IVW methods. IVW, inverse-variance weighted. OR, odds ratio.

IVW analysis identified six gut microbial genera associated with the risk of LS-BL. Protective effects against LS-BL were demonstrated by *Clostridium* innocuum group (OR = 0.841, 95%CI: 0.709–0.999), Eubacterium rectale group (OR = 0.728, 95%CI: 0.535–0.992), Anaerotruncus (OR = 0.724, 95%CI: 0.538–0.974), Ruminiclostridium6 (OR = 0.769, 95%CI: 0.601–0.984), and Lachnospiraceae UCG001 (OR = 0.801, 95%CI: 0.645–0.995). Conversely, an increased risk was associated with Lachnospiraceae UCG008 (OR = 1.282, 95%CI: 1.040–1.579) (Figure 5). Scatter plots from the MR analysis displayed the estimated effects of GM SNPs on LS-BL (Supplementary Figure 3A).

Results from Cochran’s Q, MR Egger, and MR-PRESSO analyses revealed no significant heterogeneity or horizontal pleiotropy among the six genera related to LS-BL (Table 4). Leave-one-out analysis, funnel plots, and forest plots showed no significant outliers, underscoring the reliability of the MR study results (Supplementary Figures 3B, C).

**TABLE 4:** Sensitivity analysis of the MR analysis results of the GM and LS-BL.

Exposure	Heterogeneity		Directional pleiotropy		MR-PRESSO
	Cochran’s Q	*p*-value	Egger intercept	*p*-value	*p*-value
*Clostridium* innocuum group	5.687	0.682	−0.060	0.348	0.729
Eubacterium rectale group	9.478	0.394	0.005	0.890	0.540
Anaerotruncus	9.219	0.684	−0.030	0.353	0.711
Ruminiclostridium6	16.401	0.356	−0.009	0.769	0.439
Lachnospiraceae UCG008	7.876	0.547	−0.068	0.270	0.543
Lachnospiraceae UCG001	10.270	0.672	−0.007	0.881	0.762

#### 3.2.4 Causal relationship between GM and HFNS-BL

**FIGURE 6:**
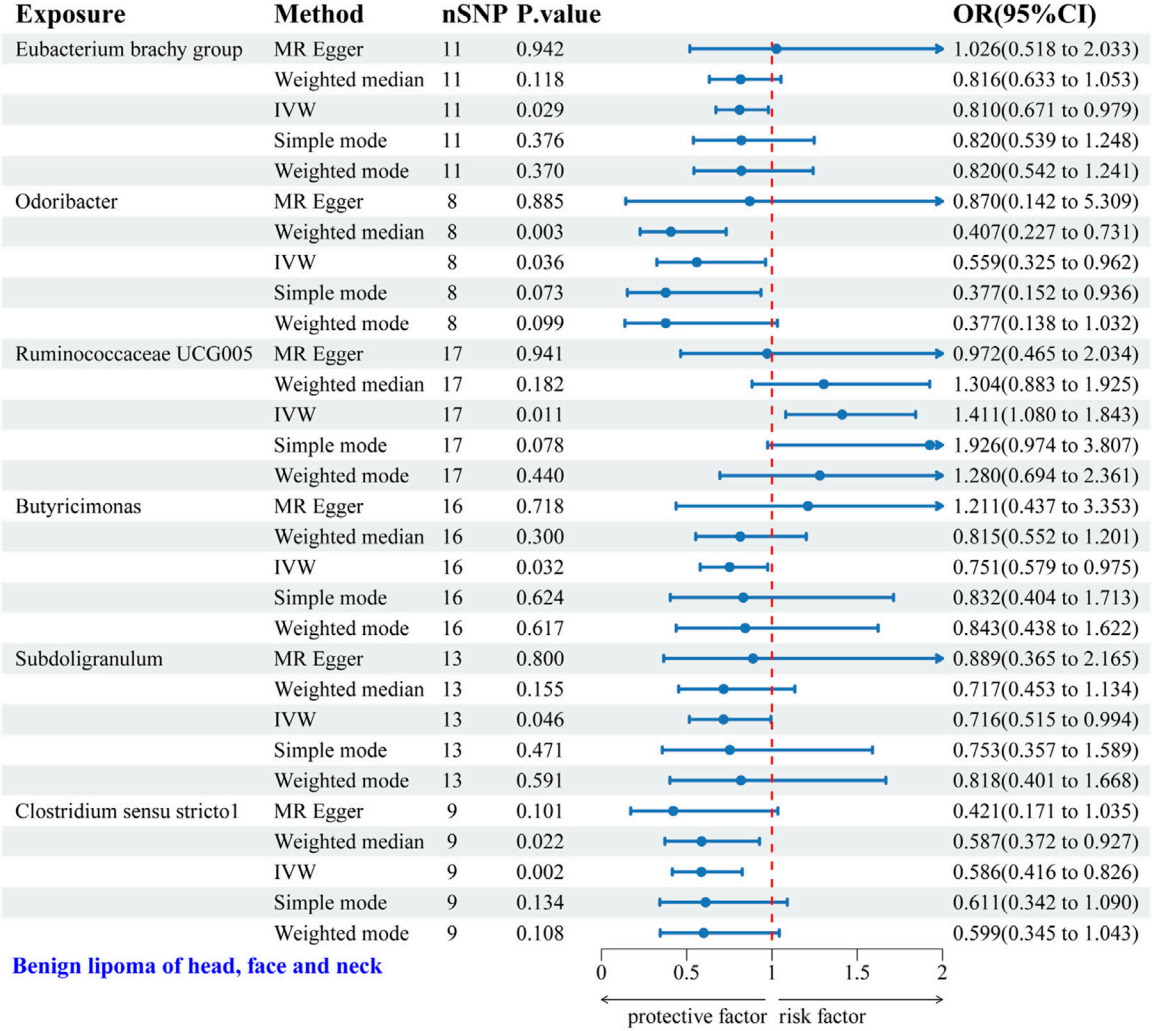
Forest plot of the associations between genetically predicted GM and HFNS-BL risk using IVW methods. IVW, inverse-variance weighted. OR, odds ratio.

IVW analysis identified six gut microbial genera associated with the risk of HFNS-BL. Protective effects were shown by Eubacterium brachy group (OR = 0.810, 95% CI: 0.671–0.979), Odoribacter (OR = 0.559, 95% CI: 0.325–0.962), Butyricimonas (OR = 0.751, 95% CI: 0.579–0.975), Subdoligranulum (OR = 0.716, 95% CI: 0.515–0.994), and *Clostridium* sensu stricto1 (OR = 0.586, 95% CI: 0.416–0.826), each indicating a protective role against HFNS-BL. Conversely, Ruminococcaceae UCG005 was associated with an increased risk (OR = 1.411, 95% CI: 1.080–1.843) (Figure 6). Scatter plots from the MR analysis displayed the estimated effects of GM SNPs on HFNS-BL (Supplementary Figure 4A).

Results from Cochran’s Q, MR Egger, and MR-PRESSO analyses indicated no significant heterogeneity or horizontal pleiotropy among the six genera related to HFNS-BL (Table 5). Leave-one-out analysis, funnel plots, and forest plots confirmed the absence of significant outliers, corroborating the reliability of the MR study results (Supplementary Figures 4B, C).

**TABLE 5:** Sensitivity analysis of the MR analysis results of the GM and HFNS-BL.

Exposure	Heterogeneity		Directional pleiotropy		MR-PRESSO
	Cochran’s Q	*p*-value	Egger intercept	*p*-value	*p*-value
Eubacterium brachy group	5.406	0.798	−0.032	0.499	0.818
Odoribacter	11.825	0.066	−0.035	0.632	0.127
Ruminococcaceae UCG005	10.849	0.763	0.033	0.305	0.758
Butyricimonas	13.999	0.450	−0.041	0.358	0.469
Subdoligranulum	9.586	0.568	−0.017	0.618	0.651
*Clostridium* sensu stricto1	7.213	0.407	0.034	0.461	0.554

## 4 Discussion

To our knowledge, supported by the GWAS database, this is the inaugural study investigating the potential causal relationship between GM and lipomas across various anatomical sites using MR, thus addressing a gap in this field from a novel perspective. This study employed MR to explore potential causal links between GM and lipomas. According to our clues, eighteen types of GM may influence the progression of lipomas, providing new directions for future treatments targeting specific GM in managing lipomas.

The GM, comprising trillions of bacteria within the gastrointestinal tract, plays critical roles, including enhancing intestinal permeability, mediating oxidative stress responses, and participating in tumor immunoregulation (Jiang and Zhang, 2024). Recent studies have established a close link between the composition and diversity of GM and various cancer-related diseases, such as renal tumors (Zhang et al., 2024), aneurysms (Qiu et al., 2024), and neuroblastoma (Zexin et al., 2024). Additionally, the relationship between the GM and the TME has attracted increasing attention. Research (Li et al., 2019) demonstrates that the microbiota affects cancer initiation, progression, and treatment response by regulating the host’s immune system. A review of previous research on GM and cancer-related diseases discusses how GM and their metabolites impact the host immune system and contribute to the development of the TME (Qiu et al., 2020). The TME, crucial for tumor growth, not only controls tumor proliferation but also supports tumor invasion, metastasis, and immune evasion (Khan et al., 2020; Zeng et al., 2016). Within the TME, the GM and its metabolites alter tumor tissues by influencing the intestinal epithelium, either promoting or hindering tumor progression. This highlights the emerging importance of the GM as a critical regulator in oncology (Erdman and Poutahidis, 2015). Moreover, existing studies show that extracellular vesicles (EV) are vital in cell-to-cell communication, especially within the TME. Extracellular vesicle long RNA (exLR) in EV exhibit stability and are rich in diagnostic information. They demonstrate high sensitivity and specificity in the early diagnosis of various cancer types, such as breast cancer (BC) and colorectal cancer (CRC) (Li et al., 2020; Li et al., 2021; Liu C. et al., 2022). exLRs can influence the behavior of tumor-infiltrating lymphocytes through immunoregulatory factors and impact tumor progression by modulating immune responses (Guo et al., 2022; Su et al., 2021).

Our MR analysis indicates that the Eubacterium rectale group, Desulfovibrio, Ruminococcus1, *Clostridium* sensu stricto1, and Lachnospiraceae UCG001 may provide protective effects against BL, while Lachnospiraceae UCG008 could be a risk factor. Within these microbial communities, the Eubacterium rectale group and Lachnospiraceae UCG001 act as protective factors against LS-BL, whereas Lachnospiraceae UCG008 heightens the risk. Desulfovibrio affords protection against TS-BL, and *Clostridium* sensu stricto1 guards against HFNS-BL.

The Eubacterium rectale group, an anaerobic Gram-positive bacterium, is prevalent in human fecal samples. While no studies directly associate this group with lipomas, research suggests its involvement in other tumor types. For instance, an animal study (Lu et al., 2022) found that the Eubacterium rectale group may prevent intestinal lymphoma by mitigating chronic inflammation and minimizing the B cell response to gut bacteria. Specifically, it prevents lymphoma by modulating the TNF-induced TLR4/MyD88/NF-κB axis, thereby reducing lymphoma incidence in Eμ-Myc mice. Moreover, treatment with 20 ng/mL Eubacterium rectale Lipopolysaccharide for 2.5 h significantly increased NF-κB expression in the nuclei of HCoEpiC and NCM460 cells (Wang et al., 2021), indicating its potential as a target for preventing both BL and LS-BL due to NF-κB’s role in tumor progression (Wullaert et al., 2011).

The TME plays a crucial role in lipoma formation. Clinical trials (Atzeni et al., 2021) have shown that Lachnospiraceae UCG001 contributes to the production of short-chain fatty acids (SCFAs), while research by Matsumoto et al. (N et al., 2021) demonstrates a strong link between Lachnospiraceae UCG008 and saturated fatty acids. These studies reveal how SCFAs and saturated fatty acids differentially influence the TME, affecting tumor development and progression. SCFAs, such as butyrate, propionate, and acetate, typically exhibit anti-inflammatory, anti-proliferative, epigenetic regulatory, and immunomodulatory effects within the TME, inhibiting tumor cell proliferation and inducing apoptosis (Dong et al., 2023; Carolin et al., 2024; Gomes et al., 2023; Tian et al., 2020). In contrast, saturated fatty acids are generally associated with negative effects in the TME, promoting inflammation, increasing tumor cell proliferation and survival, and suppressing immune functions, thus aiding tumor growth and survival (Westheim et al., 2023).

Overall, SCFAs are considered protective within the TME, while saturated acids may facilitate tumor development. In research examining skin and GM against a melanoma backdrop (Mekadim et al., 2022), *Clostridium* sensu stricto 1 was significantly linked to the TME, potentially influencing immune responses and cancer dynamics. Desulfovibrio, recognized as a protective factor in BLs, could also significantly affect the TME. Research shows that Desulfovibrio can enhance antitumor immunity within the TME (Li et al., 2024; Kim et al., 2023). Furthermore, studies have noted a correlation between Desulfovibrio concentration and Parkinson’s disease severity. Desulfovibrio produces hydrogen sulfide (H₂S), which affects cell signaling in neuronal cells at low concentrations and causes severe toxicity at higher concentrations (Murros et al., 2021).

In research exploring the causal relationship between GM and cancer (Yiwen et al., 2023), Ruminococcus1 was linked to head and neck cancer, indicating its role in tumor genesis. However, its association with lipomas warrants further experimental research. Conversely, in other conditions like non-alcoholic fatty liver disease (NAFLD), Ruminococcus1 may have exacerbated the progression of NAFLD (Zhang et al., 2023).

This study boasts several strengths. Firstly, it introduces a novel genus-level analysis of GM providing a theoretical foundation for further investigation into specific microbiota mechanisms influencing lipoma development. Secondly, leveraging genetic data from a large population sample enhances the reliability of the findings. Additionally, MR analysis helps reduce the influence of confounding factors.

However, some limitations remain. Due to stringent thresholds, some microbiota were excluded, potentially omitting critical data. The study was limited to a European population, and further research is needed to generalize these findings. Additionally, potential contamination from environmental factors was not controlled. Moreover, numerous variants with small effect sizes increase the risk of false positives. Lastly, comprehensive data on other GM are required to fully assess the causal relationship between GM and lipoma risk.

## 5 Conclusion

In summary, this study assesses the causal relationship between the GM and lipomas, identifying potentially pathogenic bacterial taxa. These findings hold significant implications for clinical practice, especially in the prevention and management of lipomas. Future studies are necessary to confirm these results through mechanistic studies and to explore potential therapeutic interventions targeting the GM.

## Data Availability

The original contributions presented in the study are included in the article/Supplementary Material, further inquiries can be directed to the corresponding authors.
