# Supplementary material for: Gut Microbiota’s role in lipoma development: evidence from mendelian randomization
Source: Front Genet. 2024 Nov 15;15:1430671. doi: 10.3389/fgene.2024.1430671 (PMC11604723; doi:10.3389/fgene.2024.1430671)
Supplement: Supplementary file 2 [file Table1.docx]

Supplementary Material



**Supplementary Figure S1.** Causal estimates of exposure (Specific GM) on BL. A): Scatter plots, the slope of each line corresponding to the estimated MR effect in different models, including the conventional IVW, Weighted median, MR-Egger, Simple mode, and Weighted mode; B): Leave-one-out stability tests, Calculate the MR results of the remaining IVs after removing the IVs one by one; C): Funnel plots; D): Forest plots.



**Supplementary Figure S2.** Causal estimates of exposure (Specific GM) on TS-BL. A): Scatter plots, the slope of each line corresponding to the estimated MR effect in different models, including the conventional IVW, Weighted median, MR-Egger, Simple mode, and Weighted mode; B): Leave-one-out stability tests, Calculate the MR results of the remaining IVs after removing the IVs one by one; C): Funnel plots; D): Forest plots.



**Supplementary Figure S3.** Causal estimates of exposure (Specific GM) on LS-BL. A): Scatter plots, the slope of each line corresponding to the estimated MR effect in different models, including the conventional IVW, Weighted median, MR-Egger, Simple mode, and Weighted mode; B): Leave-one-out stability tests, Calculate the MR results of the remaining IVs after removing the IVs one by one; C): Funnel plots; D): Forest plots.



**Supplementary Figure S4.** Causal estimates of exposure (Specific GM) on HFNS-BL. A): Scatter plots, the slope of each line corresponding to the estimated MR effect in different models, including the conventional IVW, Weighted median, MR-Egger, Simple mode, and Weighted mode; B): Leave-one-out stability tests, Calculate the MR results of the remaining IVs after removing the IVs one by one; C): Funnel plots; D): Forest plots.
